# From Presentation to Resolution: Documenting a Lobular Capillary Hemangioma of the Eyelid

**DOI:** 10.7759/cureus.71330

**Published:** 2024-10-12

**Authors:** Deepaswi Bhavsar, Tushar Agrawal, Iqra Mushtaq, Kalibo Jakhalu, Shikha Rai

**Affiliations:** 1 Ophthalmology, Dr. D. Y. Patil Medical College, Hospital and Research Centre, Dr. D. Y. Patil Vidyapeeth (Deemed to be University), Pune, IND

**Keywords:** eyelid tumor, histopathology examination, lobular capillary hemangioma, oculoplasty, ophthalmology

## Abstract

Lobular capillary hemangioma is a benign vascular tumor predominantly seen in pediatric populations. A 34-year-old male developed a reddish nodular mass on the upper eyelid of his left eye. An excisional biopsy was performed, and a histopathological examination of the specimen revealed proliferative vessels lined by increased endothelial cells, devoid of nuclear atypia. The nodular mass was subsequently diagnosed as a capillary hemangioma.

## Introduction

Lobular capillary hemangioma is a benign vascular tumor distinguished by lobular aggregates of capillary-sized blood vessels. It commonly arises in the skin and mucous membranes and is characterized by rapid growth, often presenting as a pedunculated or sessile mass that is prone to significant bleeding upon manipulation. Despite its frequent occurrence, lobular capillary hemangioma is often misdiagnosed, with accurate identification achieved in only 28-42% of cases, according to two studies. While it is considered relatively common among benign vascular tumors, specific incidence rates are not consistently documented [1].

This condition is most prevalent in children, especially infants under one year of age, although it can also manifest in adults, albeit less frequently, and exhibits a slight female predominance in pediatric cases. While commonly found in the head and neck region, its presence on the eyelid is relatively rare. Most cases are located on the face, particularly the nose and oral cavity, but it can also develop in other regions, including the eyelids. In adults, the development of lobular capillary hemangioma may be associated with trauma or irritation, often following minor injuries [2]. This report presents a case of lobular capillary hemangioma of the upper eyelid, highlighting the diagnostic challenges and management strategies involved.

## Case presentation

A male patient in his early 40s presented to the ophthalmology outpatient department with a mass on his left upper eyelid. He reported that the mass had developed two months prior and was gradually increasing in size. The patient mentioned a history of trauma during his youth but had no significant medical or surgical history, nor was he taking any medications or experiencing recent trauma.

**Figure 1:**
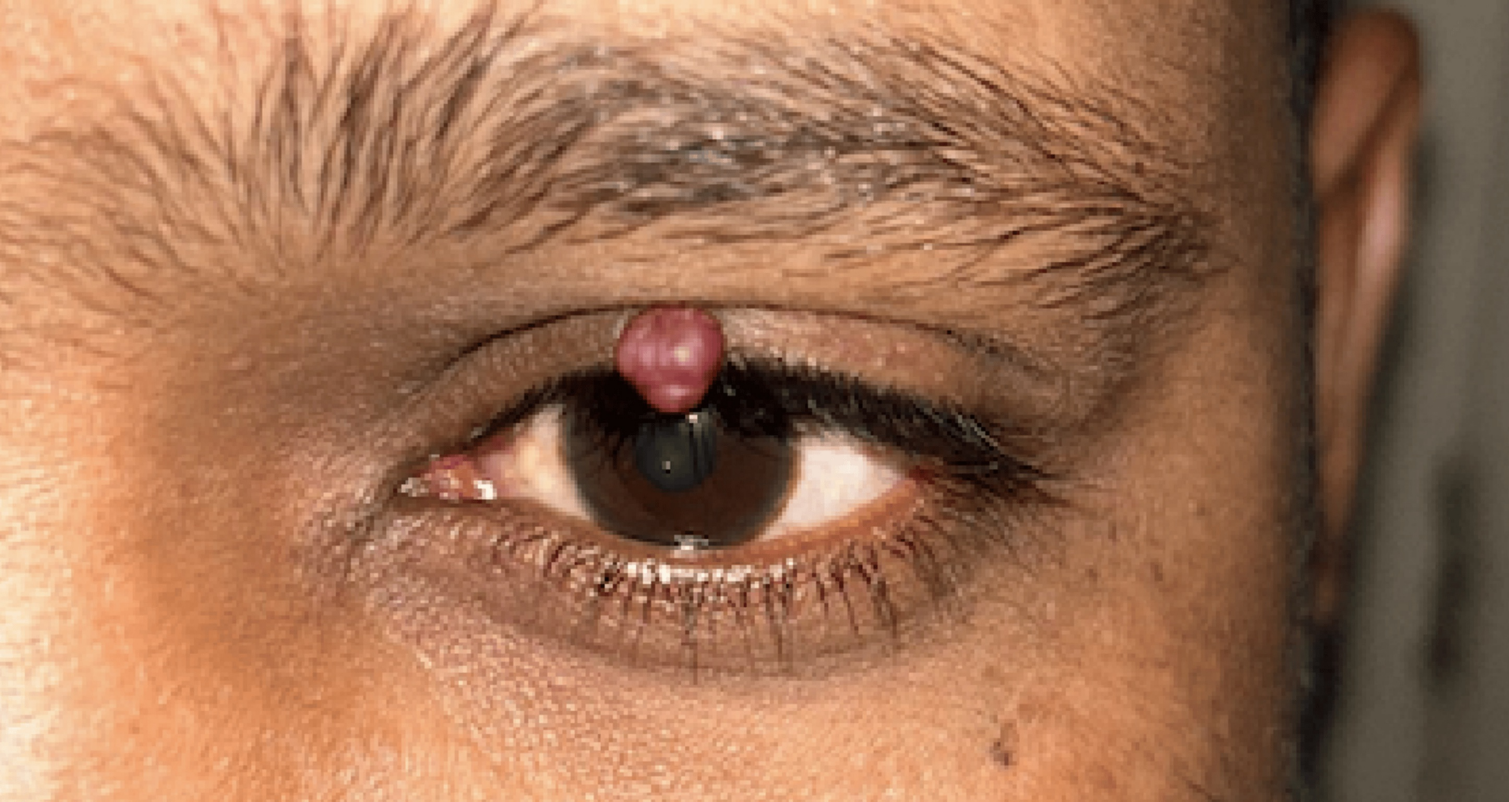
Globular mass located at the junction of the medial and lateral halves of the upper eyelid

On examination, the best corrected visual acuity (BCVA) was 20/20 in the right eye with a refractive error of -1.0 diopter sphere and 20/20 in the left eye with -1.25 diopter sphere with both eyes near vision being N6. Intraocular pressures measured by the Goldmann applanation tonometry were within normal limits. A close examination revealed a globular mass measuring 1×1×1 mm, located at the junction of the medial and lateral halves of the upper eyelid (Figure 1).

**Figure 2:**
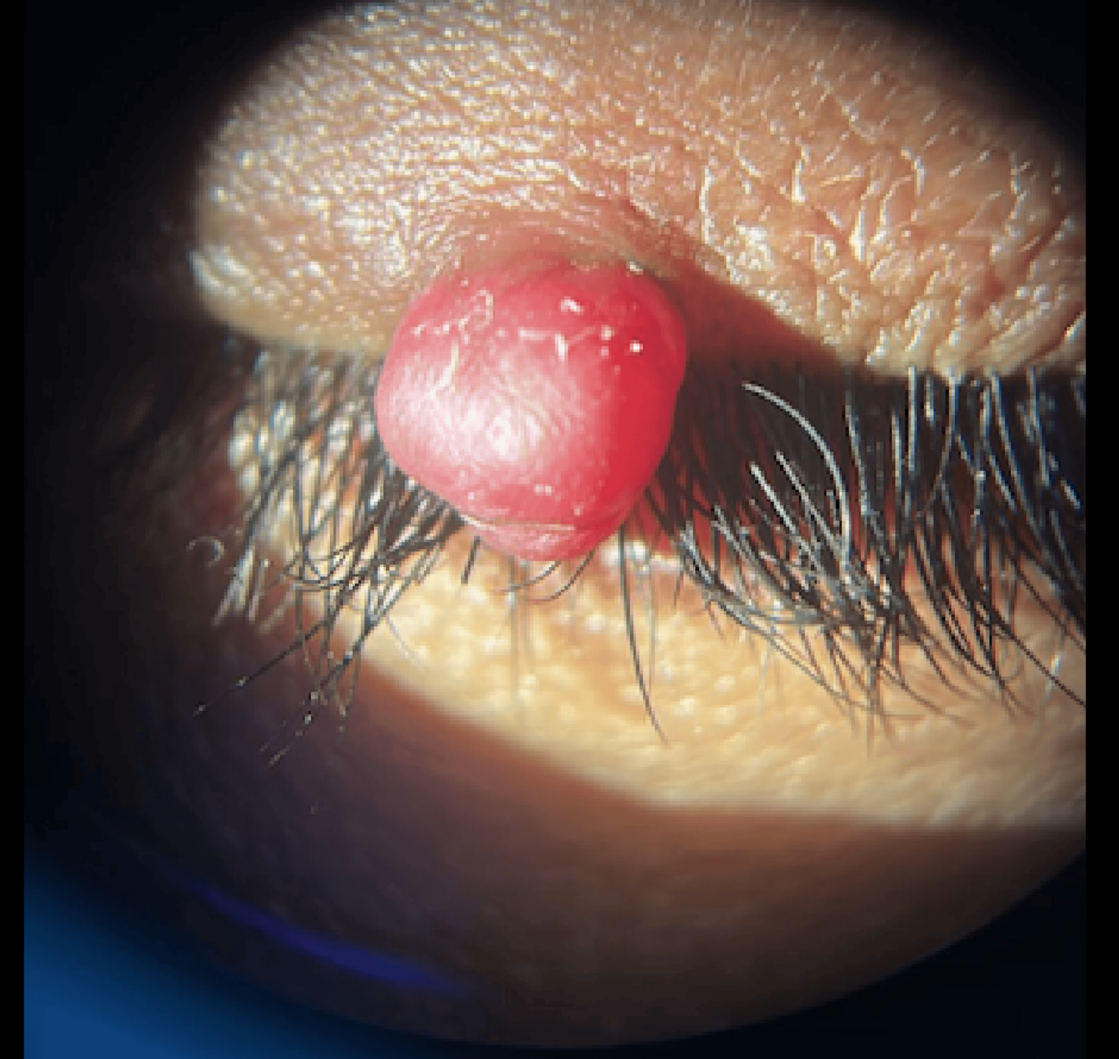
Reddish mass through high magnification measuring 1×1×1 mm

The mass was well-defined, distinctly reddish in color, and firm to the touch (Figure 2).

The anterior segment examination via slit lamp biomicroscopy was normal. A fundus examination performed with indirect ophthalmoscopy and a 20D lens showed no abnormalities or hemangiomas in either eye. No other lesions were observed elsewhere on the body. As part of the preoperative routine, a complete blood count was conducted, and all laboratory values were within normal limits.

The provisional clinical diagnosis indicated a solitary capillary hemangioma based on the characteristics of the lesion. It was concluded that an excisional biopsy was essential to ascertain the nature of the mass and to rule out any other benign or malignant conditions. The patient underwent surgical excision of the mass under local anesthesia the following month, during which the biopsy was also collected and sent for histopathological analysis. The wound was sutured using three 8-0 Vicryl stitches, and hemostasis was effectively maintained throughout the procedure.

**Figure 3:**
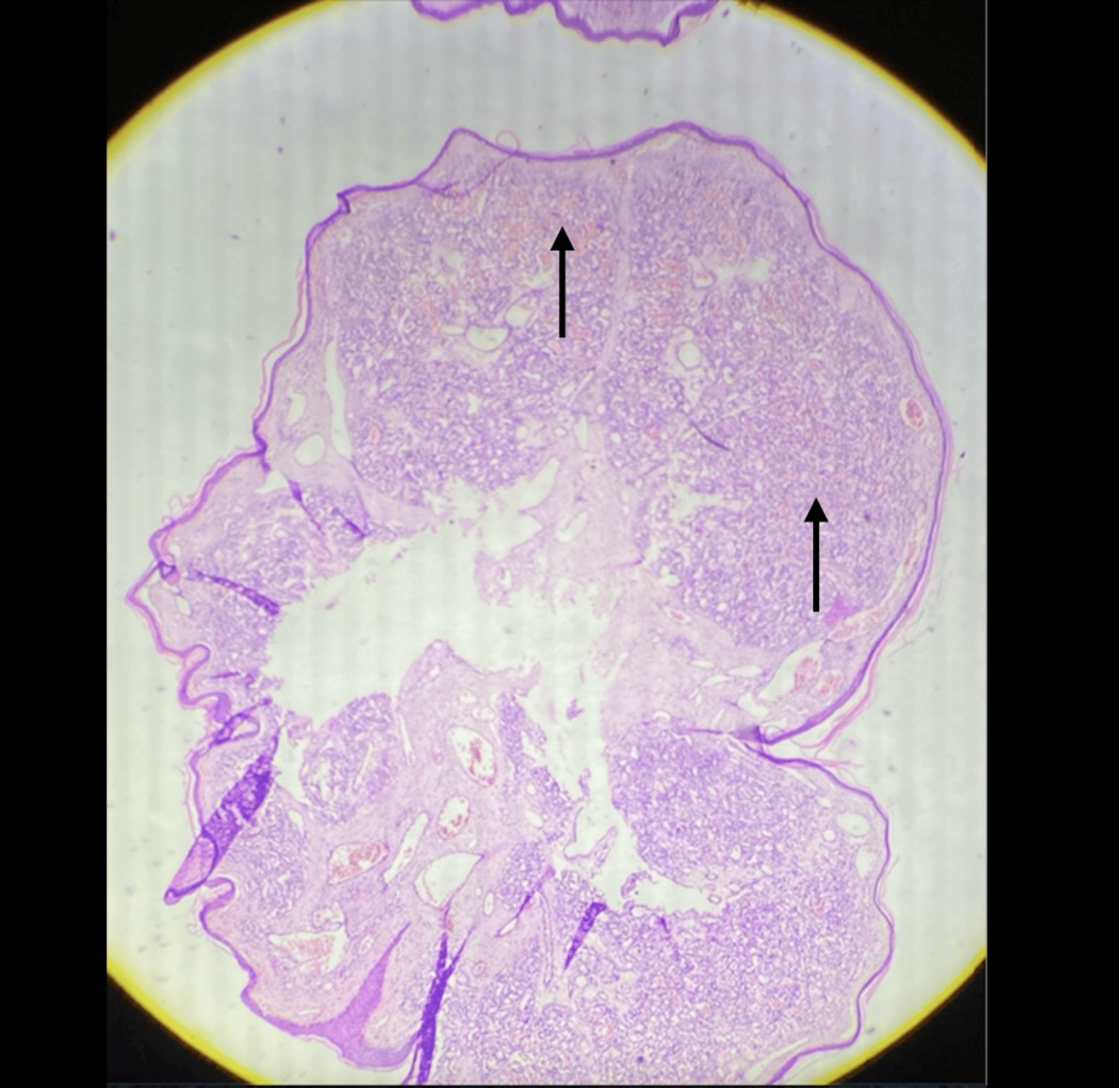
Hematoxylin and eosin-stained slide which was sent for histopathological examination Stain used: hematoxylin and eosin At 20× magnification by a Magnus microscope Arrows showing areas of lobular arrangement of proliferating capillaries with the absence of nuclear atypia

The histopathological report revealed a single piece of grey-white tissue measuring 0.5×0.4 cm, characterized by a grey-white external surface and a congested cut surface. Hematoxylin and eosin staining demonstrated small proliferating vessels arranged in a lobular configuration (Figure 3).

**Figure 4:**
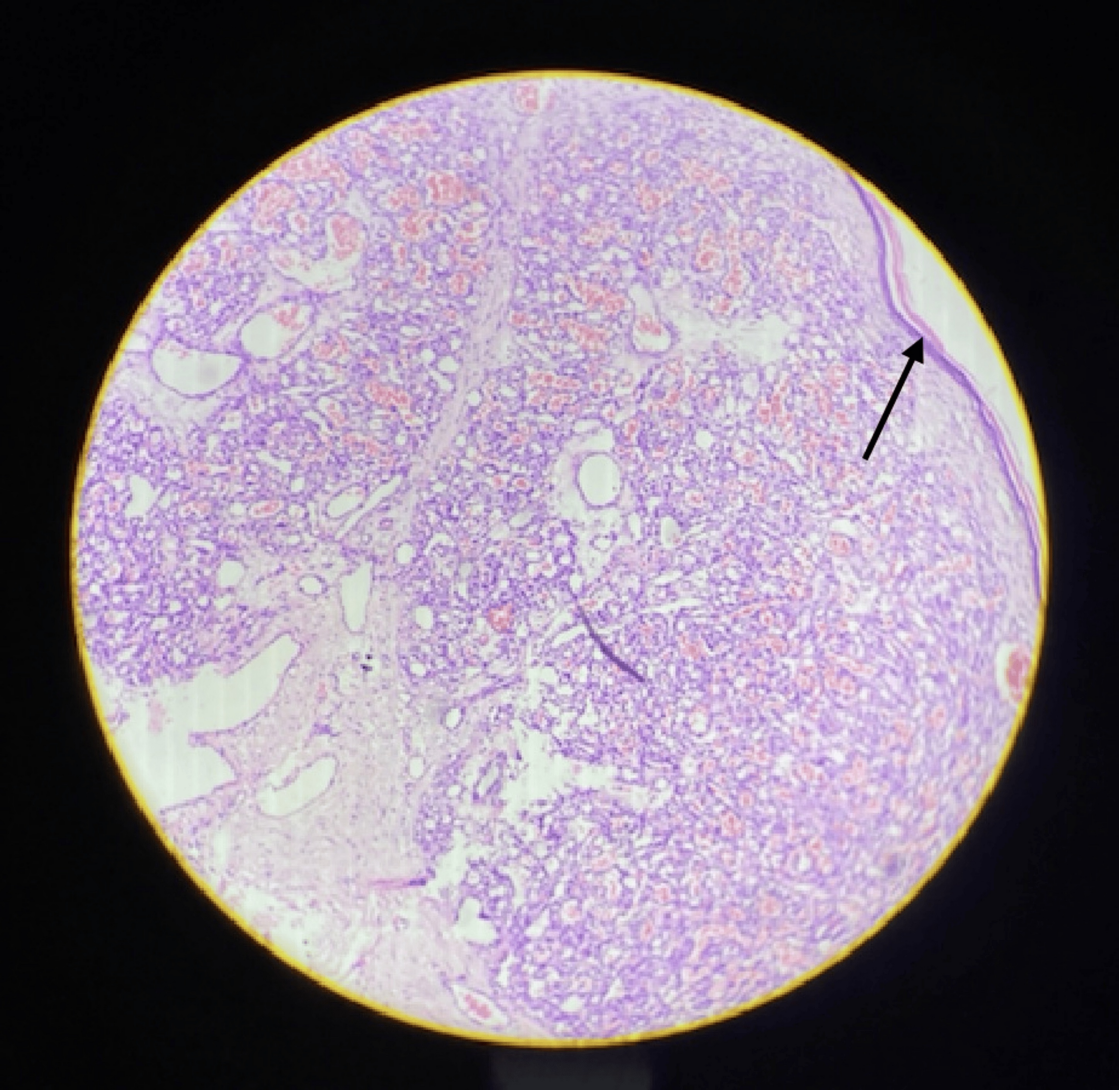
Hematoxylin and eosin staining demonstrating small proliferating vessels arranged in a lobular configuration Stain used: hematoxylin and eosin At 100× magnification by a Magnus microscope Arrow showing the fibrous capsule surrounding the lesion, as well as key cellular features with a lack of inflammation or giant cells which rules out granulomatous diseases

The overlying epidermis appeared thinned and showed focal alterations consistent with acanthosis and hyperkeratosis (Figure 4).

**Figure 5:**
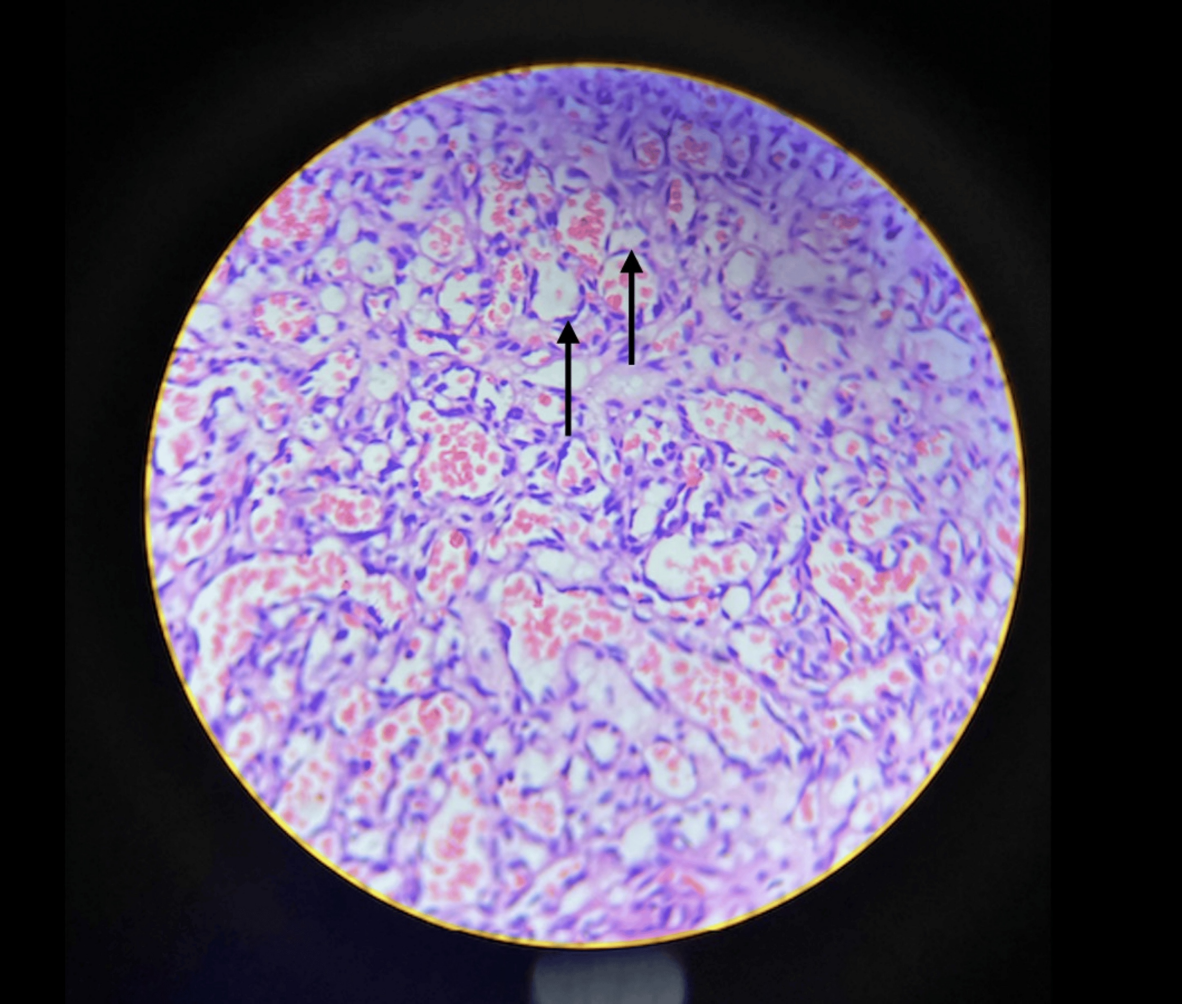
Hematoxylin and eosin-stained slide confirming the diagnosis of lobular capillary hemangioma Stain used: hematoxylin and eosin At 400× magnification by a Magnus microscope Arrows showing endothelial cells lining the capillaries and confirming the absence of nuclear pleomorphism and mitotic figures

These findings were indicative of lobular capillary hemangioma, thereby confirming the provisional diagnosis (Figure 5).

**Figure 6:**
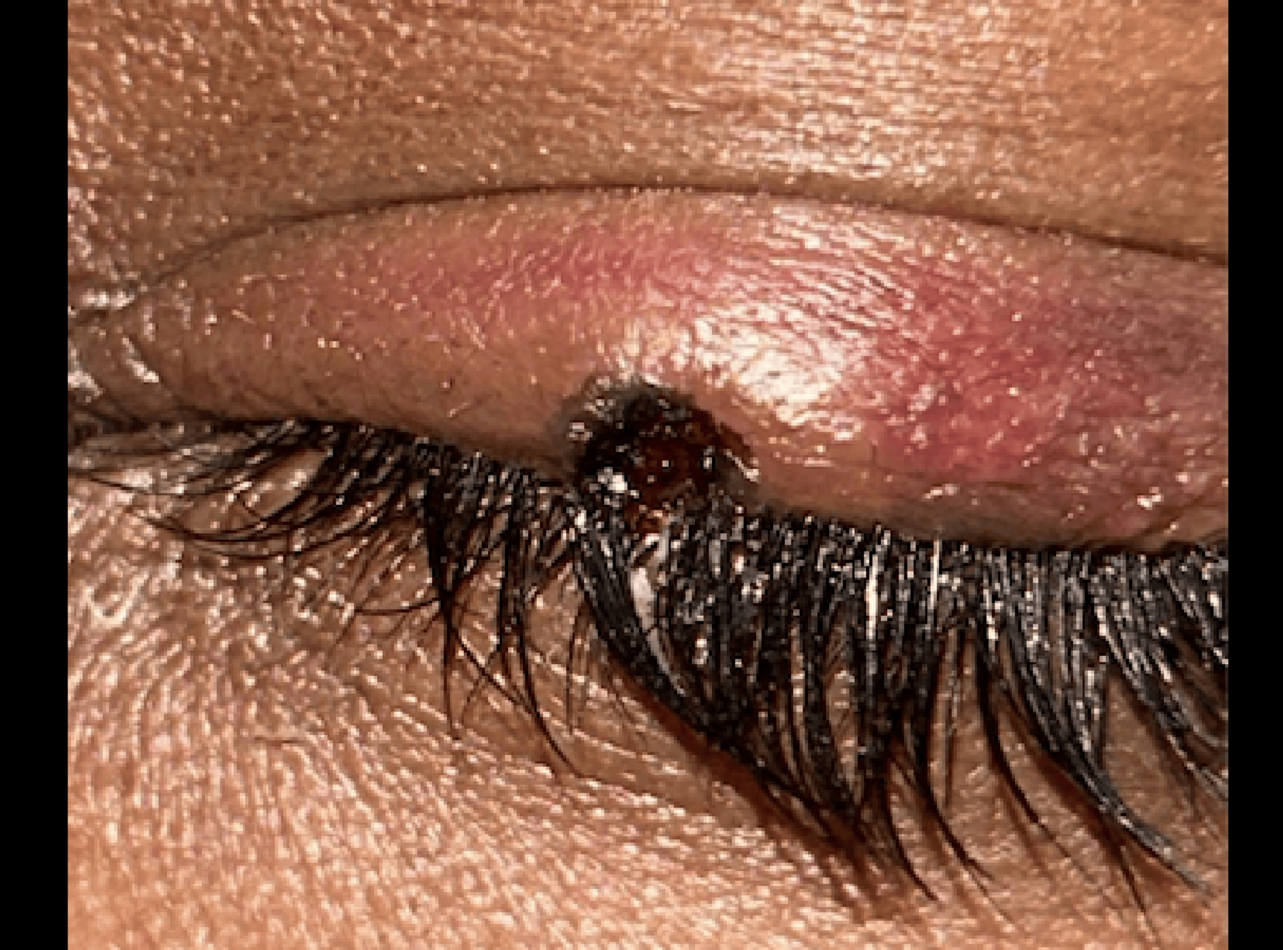
Postoperative picture after the excision of the mass Postoperative image showing the absence of swelling with no infection, recurrence, or erosion at the surgical site

The patient was assessed the day after the surgery. He was afebrile and reported no pain. The surgical site appeared clean, showing no signs of leakage, infection, redness, or swelling around the sutures (Figure 6).

He was prescribed gatifloxacin antibiotic eye drops, along with a topical ointment containing chloramphenicol, polymyxin B sulfate, and dexamethasone. At the three-week follow-up after the excision, the wound had healed satisfactorily, and both sutures were safely removed, leading to an excellent cosmetic result. Further evaluations indicated no recurrence in the eyelids or other areas of the body, and the patient had no additional concerns.

## Discussion

Lobular capillary hemangioma was initially characterized by Poncet and Dor in 1897, although reports of its occurrence in the periocular region have been infrequent [3]. In 1980, Mills introduced the term "lobular capillary hemangioma" to define a fundamental lesion associated with pyogenic granulomas. In the 2018 classification of vascular tumors by the International Society for the Study of Vascular Anomalies (ISSVA), the term pyogenic granuloma (also known as lobular capillary hemangioma) was categorized under benign vascular tumors. Diagnosis is primarily clinical, often confirmed through histopathological examination to differentiate it from other conditions. However, imaging may be essential for assessing large tumors to evaluate their vascularity and orbital extension [4].

It is thought that periocular lobular capillary hemangioma is a reactive neoplasm that develops as a result of an exaggerated healing response to local stimuli, such as irritants or minor trauma and surgical interventions. These hemangiomas can occur on the eyelid, conjunctiva, or cornea. Microscopically, they consist of a mix of acute and chronic inflammatory cells, fibroblasts, fibrocytes, and lobules of proliferating capillaries, without the presence of epithelioid giant cells, which are indicative of granulomatous inflammation [5]. Treatment typically involves surgical excision and addressing any known irritants. Small conjunctival lesions may respond to topical corticosteroids, but many will ultimately necessitate surgical intervention. In 1997, Tay et al. reported successful treatment of pediatric patients with small lobular capillary hemangiomas using pulsed dye laser therapy [6]. Surgical excision remains the most effective treatment, particularly for patients who do not respond to other therapies, which may include beta-blockers, radiotherapy, diathermy, oral steroids, and intralesional injections of bleomycin or corticosteroids. For infantile capillary hemangiomas, oral propranolol and topical timolol maleate have shown effectiveness.

It can be misinterpreted as a chalazion, which is a painless, long-standing inflammatory bump, or as basal cell carcinoma, a malignant growth that usually appears with pearly edges and possible ulceration. Furthermore, it might be confused with a papilloma, a benign growth of epithelial cells, or a pyogenic granuloma, which also appears as a fast-growing, vascular lesion often linked to injury or irritation.

Bejjanki et al. examined periocular lobular capillary hemangiomas in adults, detailing patient demographics, symptoms, and lesion characteristics. The study included histopathological analysis to distinguish these tumors from other vascular lesions, highlighting key microscopic features. The authors reviewed treatment options, such as surgical excision and follow-up, stressing the importance of accurate diagnosis to prevent mismanagement. Their work aimed to improve clinical understanding and provide practical guidance for healthcare professionals dealing with these vascular tumors [7]. Stagner and Jakobiec examined 11 adult cases of periocular lobular capillary hemangiomas, focusing on their clinical features, histopathological characteristics, and treatment responses. The authors identified diagnostic challenges due to overlapping features with other conditions and stressed the need for accurate identification. Their findings revealed variability in the behavior of these lesions and provided guidance on management strategies [8]. A common complication of orbital lesions is the development of acquired uniocular astigmatism, which can lead to amblyopia [9]. This condition may be identified at the time of initial presentation or may develop as the lesion enlarges. Thus, early diagnosis of any refractive changes is crucial for improving visual outcomes.

## Conclusions

Lobular capillary hemangiomas are common benign vascular tumors of the periocular region. While management often remains conservative due to the potential for spontaneous resolution, some cases are treated surgically for cosmetic reasons or to prevent complications, particularly when they obstruct the visual axis. Surgical options may also be pursued for earlier intervention to correct induced refractive errors, enhancing visual outcomes. For patients where surgical excision is not feasible, oral medications or intralesional injections, such as beta-blockers, corticosteroids, or cytotoxic chemotherapeutic agents, have shown effectiveness. Thus, it is essential to tailor the management approach to each patient's specific situation to effectively treat capillary hemangiomas with minimal complications.
